# MRI-based intratumoral and peritumoral radiomics predicting neoadjuvant chemotherapy response in osteosarcoma

**DOI:** 10.3389/fonc.2026.1770105

**Published:** 2026-03-13

**Authors:** Tao Zheng, Yanmiao Bai, Dabin Ren, Qirui Sui, Zhen Qian, Chuanbin Xu, Likai Wang, Kexin Zhao, Yushuang Fang, Tianran Li

**Affiliations:** 1 Clinical Medicine College, Jiamusi University, Jiamusi, Heilongjiang, China; 2 Department of Radiology, Fourth Medical Center of Chinese People’s Liberation Army (PLA) General Hospital, Beijing, China; 3 United Imaging Intelligence (Beijing), Beijing, China; 4 Department of Radiology, Taizhou Central Hospital, Taizhou Central Hospital (Taizhou University Hospital), Taizhou, Zhejiang, China; 5 Shanghai United Imaging Intelligence, Shanghai, China; 6 Health Management Service Center, Hongqi Hospital Affiliated to Mudanjiang Medical University, Mudanjiang, Heilongjiang, China

**Keywords:** intratumoral, neoadjuvant chemotherapy, nomogram, osteosarcoma, peritumoral, radiomics

## Abstract

**Objectives:**

To evaluate the predictive performance of a nomogram that integrates intratumoral and peritumoral MRI-based radiomics with clinical variables for assessing the efficacy of neoadjuvant chemotherapy (NAC) in patients with osteosarcoma (OS).

**Methods:**

This retrospective study included 93 patients with pathologically confirmed OS who underwent standard NAC. Intratumoral regions were manually segmented on axial T2-weighted fat-suppressed (T2WI-FS) images using ITK-SNAP, and peritumoral regions were generated semi-automatically by isotropic expansions of 2 mm, 4 mm, and 6 mm. Random forest classifiers were constructed separately for intratumoral, peritumoral, and combined intratumoral-peritumoral radiomics features. The optimal radiomics model was incorporated with significant clinical predictors to build an individualized nomogram. Model performance was assessed through the F1 score, Delong’s test and receiver operating characteristic (ROC) curve analysis. Decision curve analysis (DCA) was applied to assess the model’s clinical utility.

**Results:**

Multivariate logistic regression identified alkaline phosphatase (ALP) (OR = 1.003, 95% CI: 1.000 ~ 1.006, P = 0.031) and pathological fracture (PF)(OR = 2.575, 95% CI: 1.036 ~ 6.401, P = 0.042) as independent predictors of NAC response. Among all radiomics models, the Model_rad-intra + peri^2mm^ combination model demonstrated the best performance, achieving AUCs of 0.888 in the training set and 0.765 in the test set. The integrated nomogram further improved predictive accuracy, with AUC of 0.990 and 0.815 in the training and test sets, respectively.

**Conclusion:**

We developed and validated a nomogram that combines intratumoral and peritumoral MRI radiomics with clinical variables for predicting NAC efficacy in OS. The model demonstrated robust performance and may support early, individualized treatment evaluation and clinical decision-making in patients undergoing NAC.

## 1 Introduction

Osteosarcoma (OS) is the most common primary malignant bone tumor, accounting for approximately 12% of all primary bone tumors (1). It is characterized by marked aggressiveness and a high propensity for early metastasis, predominantly affecting children and adolescents (2). Previously, the 5-year survival rate of OS was low with surgical treatment alone. However, the introduction of neoadjuvant chemotherapy (NAC) has significantly improved 5-year survival (3). Despite these advances, more than half of OS patients still exhibit a poor response to NAC. The continued administration of ineffective chemotherapy regimens not only offers minimal therapeutic benefit but may also increase the risk of secondary malignancies and treatment-related toxicities (4). Therefore, accurate prediction of NAC efficacy is crucial for guiding individualized treatment strategies, optimizing clinical decision-making, and improving long-term outcomes in OS patients.

Currently, the tumor necrosis rate, which is quantified through histopathological analysis of residual viable tumor cells in postoperative specimens, is widely regarded as the gold standard for evaluating the NAC response in OS (5). However, this method has several limitations. First, it relies on postoperative pathological assessment and cannot provide early feedback during treatment. Second, dynamic monitoring of treatment response often requires repeated invasive biopsies, which carry risks of complications (6). Consequently, there is an urgent clinical need for a non-invasive, reproducible method that enables early and dynamic assessment of NAC efficacy without depending on histopathology.

Conventional MRI is routinely used to evaluate OS during NAC, primarily by assessing changes in tumor size, signal intensity, and necrotic components. Among MRI sequences, T2-weighted fat-suppressed (T2WI-FS) imaging provides high contrast between tumor tissue, bone marrow, and peritumoral edema, enabling accurate delineation of tumor extent and peritumoral changes (7). T2WI-FS was selected as the primary sequence for radiomic analysis due to its consistent acquisition across all patients, which supports feature reproducibility. Diffusion-weighted and post-contrast sequences were excluded due to incomplete availability and a higher degree of acquisition heterogeneity, which might introduce selection bias. However, the interpretation of traditional imaging features is highly dependent on the experience and subjective judgment of the imaging physician, which may lead to a decrease in the consistency of diagnosis and efficacy assessment. A recent systematic review by Zhang et al. (8) confirmed that conventional MRI parameters alone are insufficient for reliably predicting chemotherapy response in osteosarcoma. Therefore, there is an urgent need for an imaging assessment method that is objective, reproducible, and capable of quantitative analysis to compensate for the limitations of subjective qualitative analysis.

Radiomics enables high-throughput quantitative characterization of tumor phenotypes through extraction of multidimensional features from medical imaging data. By quantifying subtle variations in MRI signal intensity, texture, shape, and spatial heterogeneity, radiomics can capture microstructural and microenvironmental characteristics corresponding to underlying histopathological and molecular alterations that are imperceptible through conventional visual assessment. MRI-based radiomics has demonstrated robust predictive value across diverse oncologic applications, including treatment response prediction in liver cancer, lung cancer, rectal cancer and breast cancer (9), the noninvasive prediction of histopathology and biochemical recurrence after prostate cancer (10), and predicting the histological grade of soft tissue sarcomas (11). These advances suggest that radiomics can effectively capture intratumoral biological features associated with chemotherapeutic sensitivity. Consequently, quantitative radiomics analysis of MRI in OS may provide a non-invasive, objective biomarker for early prediction of NAC efficacy.

Most current OS radiomics studies primarily focus on intratumoral features, potentially underestimating the prognostic value of the peritumoral region. Recent work has highlighted peritumoral radiomics as a useful surrogate for tumor biological behavior (12, 13). Although radiomics has demonstrated potential in the diagnosis, prognosis, and response prediction of OS, including models based on DWI for predicting the response to NAC (14, 15), most approaches still solely characterize the intratumoral compartment. Biologically, the peritumoral zone is enriched with inflammatory infiltration, neovascularization, extracellular matrix remodeling, and invasive tumor fronts, which may influence drug delivery, hypoxia, and chemotherapy sensitivity, thereby affecting response, local recurrence, and distant metastasis. For instance, in HER2-positive breast cancer, peritumoral radiomic features have been shown to correlate with specific molecular subtypes and pathological complete response to targeted therapy, partly through their association with spatial lymphocyte distribution (16). Consistent with this rationale, studies in other malignancies suggest that integrating intra- and peritumoral radiomics improves predictive performance compared with intratumoral analysis alone (17, 18). Therefore, combining peritumoral radiomic features with intratumoral characteristics may provide a more comprehensive and clinically useful assessment of chemosensitivity in OS.

However, despite the biological relevance of the peritumoral microenvironment in OS, MRI-based intratumoral and peritumoral radiomics have not yet been systematically investigated in the context of NAC response. Addressing this gap may provide new imaging biomarkers for early, non-invasive evaluation of treatment efficacy.

## 2 Materials and methods

### 2.1 Patients

This retrospective study included 93 patients with histologically confirmed OS who received NAC at Fourth Medical Center of Chinese PLA General Hospital between January 2021 and April 2025. Patients were randomly split into a training set (n=75) and a test set (n=18) in an approximate 4:1 ratio. Five-fold cross-validation was applied within the training cohort for hyperparameter optimization and internal validation. The NAC regimen consisted of 4–6 cycles of adriamycin (ADM), high-dose methotrexate (MTX), cisplatin (DDP), and ifosfamide (IFO). Drug dosages followed National Comprehensive Cancer Network (NCCN) guidelines, with ADM 90 mg/m^2^ every 3 weeks, DDP 120–140 mg/m^2^ every 2 weeks, IFO 15 g/m^2^ every 2 weeks, and MTX 8–10 g/m^2^ every 3 weeks (19, 20).

Inclusion criteria were: (I) histopathologically confirmed OS; (II) MRI performed before initiation of NAC; (III) completion of the full planned NAC regimen; and (IV) definitive surgical resection after NAC. Exclusion criteria included: (I) history of other malignancies; (II) inadequate MRI image quality; (III) prior treatments other than NAC; and (IV) incomplete clinical, pathological, or imaging data.

**Figure 1:**
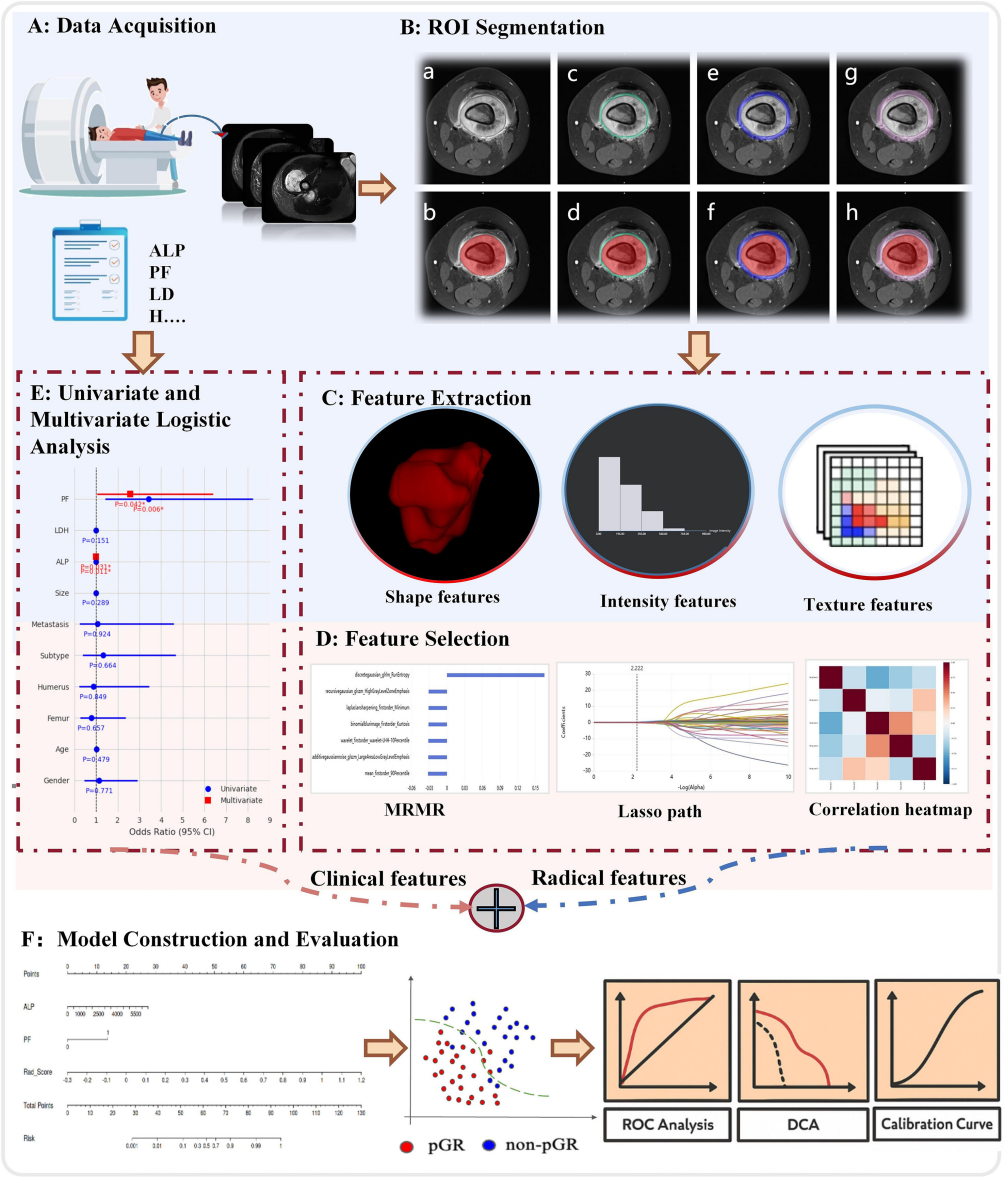
The radiomics workflow. **(A)** Data acquisition including MRI imaging and clinical variables. **(B)** ROI segmentation and generation of peritumoral regions: (a) original image; (b) intratumoral ROI; (c) peritumoral^2mm^; (d) intratumoral + peritumoral ^2mm^; (e) peritumoral^4mm^; (f) intratumoral + peritumoral^4mm^; (g) peritumoral^6mm^; (h) intratumoral + peritumoral^6mm^. **(C)** Feature extraction including shape, intensity, and texture features. **(D)** Feature selection using MRMR, LASSO, and correlation analysis. **(E)** Statistical analysis. **(F)** Model evaluation including ROC analysis, decision curve analysis, and calibration curves.

Baseline patient information included age, sex, primary tumor location, histological subtype, maximum tumor diameter, presence of pathological fracture (PF), pulmonary metastasis status at diagnosis, serum ALP level, serum lactate dehydrogenase (LDH) level, and chemotherapy response. Serum ALP and LDH were routinely measured in all patients as part of the standard laboratory workup at our institution. PF was diagnosed based on available imaging (radiography, MRI, or CT) and clinical symptoms. Extremity CT was not routinely performed and was only obtained when other imaging was inconclusive. According to established criteria (21), postoperative pathological response was assessed by the percentage of tumor necrosis in the resected specimen, with ≥90% necrosis classified as a pathological good response (pGR) and<90% as non-pGR. All pathological evaluations were performed by experienced pathologists blinded to imaging data. The overall workflow of this study is illustrated in **Figure 1**.

### 2.2 MRI image acquisition and preprocessing

All patients underwent pre-treatment MRI examinations that included T2-weighted fat-suppressed (T2WI-FS) sequences, acquired using Siemens Magnetom Trio 1.5T. Key acquisition parameters for T2WI-FS included a repetition time (TR) of 2000 ms, echo time (TE) of 90 ms, slice thickness of 5 mm, matrix size of 384×384. To minimize scanner and protocol-related variability, all MRI datasets underwent a standardized preprocessing workflow prior to radiomic feature extraction. The pipeline consisted of the following steps: (I) intensity normalization using Z-score standardization to harmonize grayscale distributions across scanners, computed as:


$$I_{normalized}=\frac{I-u}{\sigma }$$

where μ and σ represent the mean and standard deviation of voxel intensities, respectively; (II) resampling all images to an isotropic voxel size of 1×1×3 mm³ using B-spline interpolation to reduce anisotropic spatial bias; and (III) gray-level discretization with a fixed bin width in accordance with IBSI recommendations to improve the robustness and reproducibility of texture features.

### 2.3 Intratumoral and peritumoral ROI annotation

Tumor segmentation was conducted on T2-weighted fat-suppressed (T2WI-FS) images using ITK-SNAP software (www.itk-snap.org). Two experienced radiologists manually segmented the intratumoral region of interest (ROI) slice-by-slice on axial images, and all segmentations were reviewed by a senior radiologist with over 20 years of experience. To assess inter-observer consistency, one radiologist repeated the segmentation for a randomly selected subset of 30 patients after a one-week interval. This sample size was chosen based on established recommendations in radiomics methodology, where 20–30 cases are generally sufficient for stable ICC estimation. Peritumoral ROIs were generated by applying three-dimensional morphological dilation to the intratumoral ROI using the uAI Research Portal (version 20250130sp1, https://urp.united-imaging.com), with expansion distances of 2 mm, 4 mm, and 6 mm. All radiologists were blinded to clinical and pathological outcomes throughout the segmentation and processing procedures. To assess interobserver reliability of tumor segmentation, intraclass correlation coefficients (ICCs) were calculated based on radiomic features extracted from a randomly selected subset of 30 patients. An ICC greater than 0.75 was considered indicative of good agreement, and an ICC greater than 0.90 indicated excellent agreement.

### 2.4 Feature extraction and selection

Radiomic features were extracted from all seven ROIs using the United Imaging Research platform uAI Research Portal, which computes first-order, shape, and texture features in accordance with IBSI guidelines. Features were derived from seven predefined ROIs, including the intratumoral ROI, three peritumoral ROIs expanded by 2 mm, 4 mm, and 6 mm (peritumoral^2mm^ ROI, peritumoral^4mm^ ROI, peritumoral^6mm^ ROI), and three combined ROIs that integrated intratumoral and peritumoral regions (intratumoral + peritumoral^2mm^ ROI, intratumoral + peritumoral^4mm^ ROI, intratumoral + peritumoral ^6mm^ ROI). Features were extracted from both original images and filtered images (including wavelet and LoG-transformed representations), covering first-order statistics, shape descriptors, texture matrices (GLCM, GLRLM, GLSZM), and higher-order features. To eliminate non-informative features, variance thresholding was first applied to remove features with zero variance, followed by Z-score normalization to improve feature comparability. Minimum Redundancy Maximum Relevance (mRMR) was then used to identify features with high predictive relevance and low redundancy. Subsequently, least absolute shrinkage and selection operator (LASSO) regression with L1 regularization was applied, with the optimal penalty parameter λ determined via cross-validation to minimize the mean squared error. This procedure shrank coefficients of low-contributing features toward zero and retained the most predictive subset. A radiomics score (Rad-score) was finally computed as a linear combination of the selected features weighted by their LASSO coefficients, providing a quantitative index of the association between imaging phenotypes and NAC response.

### 2.5 Model construction and evaluation

#### 2.5.1 Radiomics model construction

Seven radiomics models were developed using a random forest classifier based on the selected optimal radiomic feature sets. The models consisted of: (I) an intratumoral radiomics model (Model_rad–intra); (II) three peritumoral radiomics models constructed using different expansion distances (Model_rad-peri^2mm^, Model_rad-peri^4mm^, and Model_rad-peri^6mm^); and (III) three combined intratumoral – peritumoral radiomics models (Model_rad-intra+peri^2mm^, Model_rad-intra+peri^4mm^, and Model_rad-intra+peri^6mm^). Hyperparameters were optimized using five-fold cross-validation coupled with a grid-search procedure to determine the optimal configuration for each classifier. For each radiomics model, the prediction probability generated by the random forest classifier was used as the radiomics score (Rad-score) to quantitatively evaluate individual response to NAC.

#### 2.5.2 Clinical model construction

Univariate and multivariate logistic regression analyses were conducted to assess the associations between clinical variables and pGR. Variables with p< 0.1 in the univariate analysis were entered into the multivariate model to identify independent predictors of pGR. A clinical prediction model was subsequently constructed based on these independently significant predictors.

#### 2.5.3 Construction of the nomogram and SHAP interpretive analysis method

To evaluate the predictive performance of the radiomics models, receiver operating characteristic (ROC) curves and their corresponding areas under the curve (AUCs) were calculated for each model, along with accuracy, sensitivity, and specificity. In addition, the F1-score was reported to provide a balanced measure of model performance by jointly considering precision and recall, particularly in the presence of potential class imbalance between the pGR and non-pGR groups. The model with the best overall performance was selected to construct the integrated prediction model (Model_rad-combined). Based on this integrated model, a nomogram and a Shapley additive explanations (SHAP) summary plot were generated to enhance model interpretability and facilitate clinical application. To further assess the clinical utility of each model, decision curve analysis (DCA) was performed to quantify the net benefit across a range of threshold probabilities. Additionally, calibration curves were plotted to evaluate the agreement between predicted and observed probabilities, thereby examining both the calibration performance and practical reliability of the Model_rad-combined.

### 2.6 Statistical analysis

Statistical analyses were performed using SPSS software (IBM SPSS Statistics, IBM Corp., Armonk, NY, USA). Continuous variables were expressed as means ± standard deviations (SD), whereas categorical variables were summarized as frequencies and percentages. The normality of continuous variables was assessed using the Kolmogorov–Smirnov test. For normally distributed variables, comparisons between groups were performed using the independent-samples *t* test, whereas the Mann–Whitney *U* test was applied for non-normally distributed variables. Categorical variables were compared using the chi-square test or Fisher’s exact test, as appropriate. Delong’s test was applied to evaluate significant differences between AUCs. Finally, decision curve analysis (DCA) was performed to assess the model’s clinical utility. *P* < 0.05 represented statistical significance.

## 3 Results

### 3.1 Patient characteristics

A total of 93 patients were included in this study and randomized into a training set (n = 75) and a test set (n = 18). Patients were classified into a pGR group (n = 45) and a non-pGR group (n = 48) based on postoperative pathological assessment. Analysis of baseline clinical characteristics revealed potential differences in ALP levels and the presence of PF between the pGR and non-pGR groups (P< 0.1), as summarized in **Table 1**. Specifically, the distribution of ALP showed a trend toward difference between the two groups in both the training and test sets (P< 0.1). PF demonstrated a potential association with pathological response in the training set (P< 0.1), whereas no statistically significant difference was observed in the test set (P ≥ 0.1), likely due to the smaller sample size. No statistically significant differences (P ≥ 0.05) were observed in other variables, including age, sex, tumor location, pathological subtype, maximum tumor diameter, presence of distant lung metastasis, LDH level, or other clinical factors in either the training or test sets, as detailed in **Table 2**.

**Table 1:** Baseline characteristics of the study sample.

Variables	Total (n=93)	pGR (n=45)	non-pGR (n=48)	*t/Z/χ^2^*	P value
Sex				0.084	0.771
Male	24(25.8)	11(24.4)	13(27.1)		
Female	69(74.2)	34(75.6)	35(72.9)		
Age	17.13 ± 9.01	16.44 ± 8.60	17.77 ± 8.89	-0.558	0.557
Location				0.212	0.899
Femur	60(64.5)	30(66.7)	30(62.4)		
Tibia	17(18.3)	8(17.8)	8(18.8)		
Others	16(17.2)	7(15.5)	7(18.8)		
Diameter	100.24 ± 39.56	104.58 ± 43.34	96.17 ± 35.63	-0.596	0.551
Pathological fracture				7.859	0.005
None	36(38.7)	24(53.3)	12(25)		
Yes	57(61.3)	21(46.7)	36(75)		
Lung metastasis				0.009	0.924
None	85(91.4)	41(91.1)	44(91.7)		
Yes	8(8.6)	4(8.9)	4(8.4)		
Pathological subtypes				0.189	0.663
Common	82(88.2)	39(86.7)	43(89.6)		
Others	11(11.8)	6(13.3)	5(10.4)		
ALP	341.49 ± 648.38	207.00 ± 121.98	467.58 ± 880.58	-2.845	0.004
LDH	213.97 ± 75.55	225.73 ± 88.60	202.94 ± 59.71	-0.826	0.409

ALP, alkaline phosphatase; LDH, lactate dehydrogenase.

**Table 2:** Comparison of clinicopathological data of OS patients in the training set and test set.

Variables	Training set (n=75)				Test set (n=18)			
	pGR (n=36)	non-pGR (n=39)	*t/Z/χ^2^*	P value	pGR (n=9)	non-pGR (n=9)	*t/Z/χ^2^*	P value
Sex								
Male	10(27.8)	10(25.6)	0.044	0.834	1(11.1)	3(33.3)	1.286	0.257
Female	26(72.2)	29(74.4)			8(88.9)	6(66.7)		
Age	16.97 ± 9.32	17.82 ± 9.77	-0.223	0.823	14.33 ± 4.58	17.56 ± 8.31	-0.355	0.723
Location								
Femur	25(69.4)	26(66.7)	0.684	0.710	2(55.6)	4(44.4)	1.111	0.574
Tibia	6(16.7)	5(12.8)			2(22.2)	4(44.4)		
Others	5(13.9)	8(20.5)			2(22.2)	1(11.2)		
Diameter	100.69 ± 44.35	94.67 ± 36.21	-0.217	0.828	120.11 ± 37.29	102.67 ± 33.67	-0.927	0.354
Pathological fracture								
None	20(55.6)	11(28.2)	5.775	0.016	4(44.4)	1(11.1)	2.492	0.114
Yes	16(44.4)	28(71.8)			5(55,6)	8(88.9)		
Lung metastasis								
None	34(94.4)	35(89.7)	0.562	0.453	7(77.8)	9(100)	2.250	0.134
Yes	2(5.6)	4(10.3)			2(22.2)	0(0)		
Pathological subtypes								
Common	31(86.1)	36(92.3)	0.754	0.385	8(88.9)	7(77.8)	0.400	0.527
Others	5(13.9)	3(7.7)			1(11.1)	2(22.2)		
ALP	201.89 ± 124.34	453.05 ± 964.56	-2.264	0.024	227.44 ± 116.70	530.56 ± 361.69	-2.075	0.038
LDH	221.92 ± 76.35	206.13 ± 58.04	-0.578	0.563	241.00 ± 131.71	189.11 ± 68.42	-0.927	0.354

ALP, alkaline phosphatase; LDH, lactate dehydrogenase.

### 3.2 Feature selection

**Figure 2:**
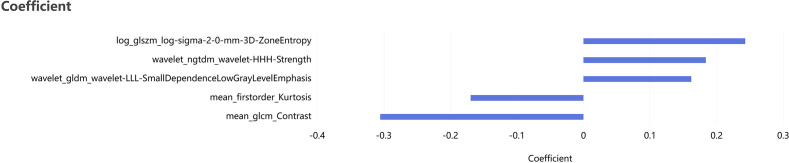
The optimal radiomics features and their weights of the intratumoral + peritumoral^2mm^ selected by LASSO regression (LASSO: 0.00599).

A total of seven ROIs were generated, including the intratumoral region, three peritumoral regions (expanded by 2 mm, 4 mm, and 6 mm), and three combined intratumoral–peritumoral regions. For each ROI, 2,264 radiomics features were extracted. Following feature screening and dimensionality reduction, the optimal feature subsets associated with pGR prediction were obtained, comprising 7, 8, 11, 8, 5, 6, and 6 features, respectively. **Figure 2** presents the feature selection pathway and the coefficient profiles of the LASSO regression model for Model_rad-intra+peri^2mm^, with the optimal regularization parameter (λ) determined as 0.00599. Interobserver reliability analysis showed good to excellent agreement for tumor segmentation, with a mean ICC of 0.88 (range, 0.82–0.94) across the selected radiomic features.

### 3.3 Construction of the radiomic model

Seven radiomics models were developed to distinguish postoperative pGR status. Their predictive performance was evaluated using the area under the curve (AUC), accuracy, sensitivity, specificity, and F1 score in both the training and test sets (**Table 3**). Overall, the radiomics models demonstrated robust performance in differentiating postoperative pGR status. Specifically, Model_rad-intra+peri^2mm^ achieved an AUC of 0.765 in the test set, outperforming Model_rad-intra (AUC = 0.728) and Model_rad-peri^2mm^ (AUC = 0.744), as well as other peritumoral and combined-region models. Based on its superior performance, the radiomics score (rad-score) from Model_rad-intra+peri^2mm^ was selected for further analysis.

**Table 3:** Efficacy comparison of different intratumoral and peritumoral radiomics models.

Model	AUC (95%CI)	Accuracy	Sensitivity	Specificity	Precision	F1
Training set						
Intratumoral	0.927(0.871-0.983)	0.840	0.769	0.917	0.909	0.833
peritumoral ^2mm^	0.965(0.931-0.999)	0.919	0.974	0.861	0.881	0.925
peritumoral ^4mm^	0.977(0.947-1.000)	0.946	0.947	0.944	0.947	0.947
peritumoral ^6mm^	0.919(0.858-0.980)	0.811	0.947	0.667	0.837	0.837
Intratumoral + peritumoral ^2mm^	0.888(0.813-0.963)	0.747	0.641	0.861	0.833	0.725
Intratumoral + peritumoral ^4mm^	0.977(0.947-1.000)	0.919	0.974	0.861	0.881	0.925
Intratumoral + peritumoral ^6mm^	0.945(0.896-0.994)	0.827	0.974	0.667	0.760	0.854
Test set						
Intratumoral	0.728(0.471-0.986)	0.667	0.778	0.556	0.636	0.700
peritumoral ^2mm^	0.744(0.507-0.982)	0.684	0.800	0.556	0.667	0.727
peritumoral ^4mm^	0.511(0.228-0.794)	0.474	0.500	0.444	0.500	0.500
peritumoral ^6mm^	0.600(0.313-0.887)	0.632	0.700	0.556	0.636	0.667
Intratumoral + peritumoral ^2mm^	0.765(0.528-1.000)	0.667	0.889	0.444	0.615	0.727
Intratumoral + peritumoral ^4mm^	0.522(0.240-0.804)	0.579	0.700	0.444	0.583	0.636
Intratumoral + peritumoral ^6mm^	0.741(0.477-1.000)	0.722	0.667	0.778	0.750	0.706

AUC, area under curve.

### 3.4 Clinical model construction and evaluation

Univariate and multivariate logistic regression analyses were performed to evaluate the association between clinical variables and pGR. Predictors with a p-value< 0.1 in the univariate analysis were included in the multivariate model to identify independent factors associated with pGR. A clinical model was subsequently constructed based on the identified independent predictors (Model_clinical). The results are shown in **Table 4**.

**Table 4:** Results of univariate and multivariate logistic regression analyses.

Characteristic	Univariate analysis		Multivariate analysis	
	OR(95%Cl)	P value	OR(95%Cl)	P value
Sex	1.148(0.452-2.914)	0.771	–	–
Age	1.017(0.971-1.065)	0.479	–	–
Location		–	–	–
Femur	0.778(0.256-2.360)	0.657	–	–
Tibia	0.875(0.222-3.451)	0.849	–	–
Others		–	–	–
Pathological subtypes	1.323(0.374-4.681)	0.664	–	–
Lung metastasis	1.073(0.252-4.574)	0.924	–	–
Diameter	0.994(0.984-1.005)	0.289	–	–
ALP	1.004(1.001-1.007)	0.011	1.003(1.000-1.006)	0.031
LDH	0.996(0.990-1.002)	0.151	–	–
Pathological fracture	3.429(1.426-8.244)	0.006	2.575(1.036-6.401)	0.042

OR, odds ratio; CI, confidence interval.

### 3.5 Combined model construction and evaluation

**Figure 3:**
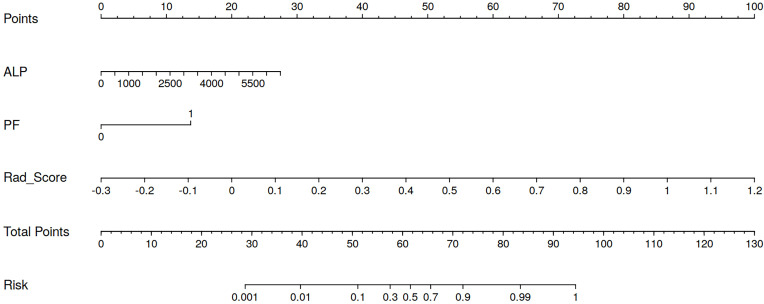
Nomogram prediction model constructed by combining intratumoral + peritumoral ^2mm^ radiomics scores with clinicopathological features.

**Figure 4:**
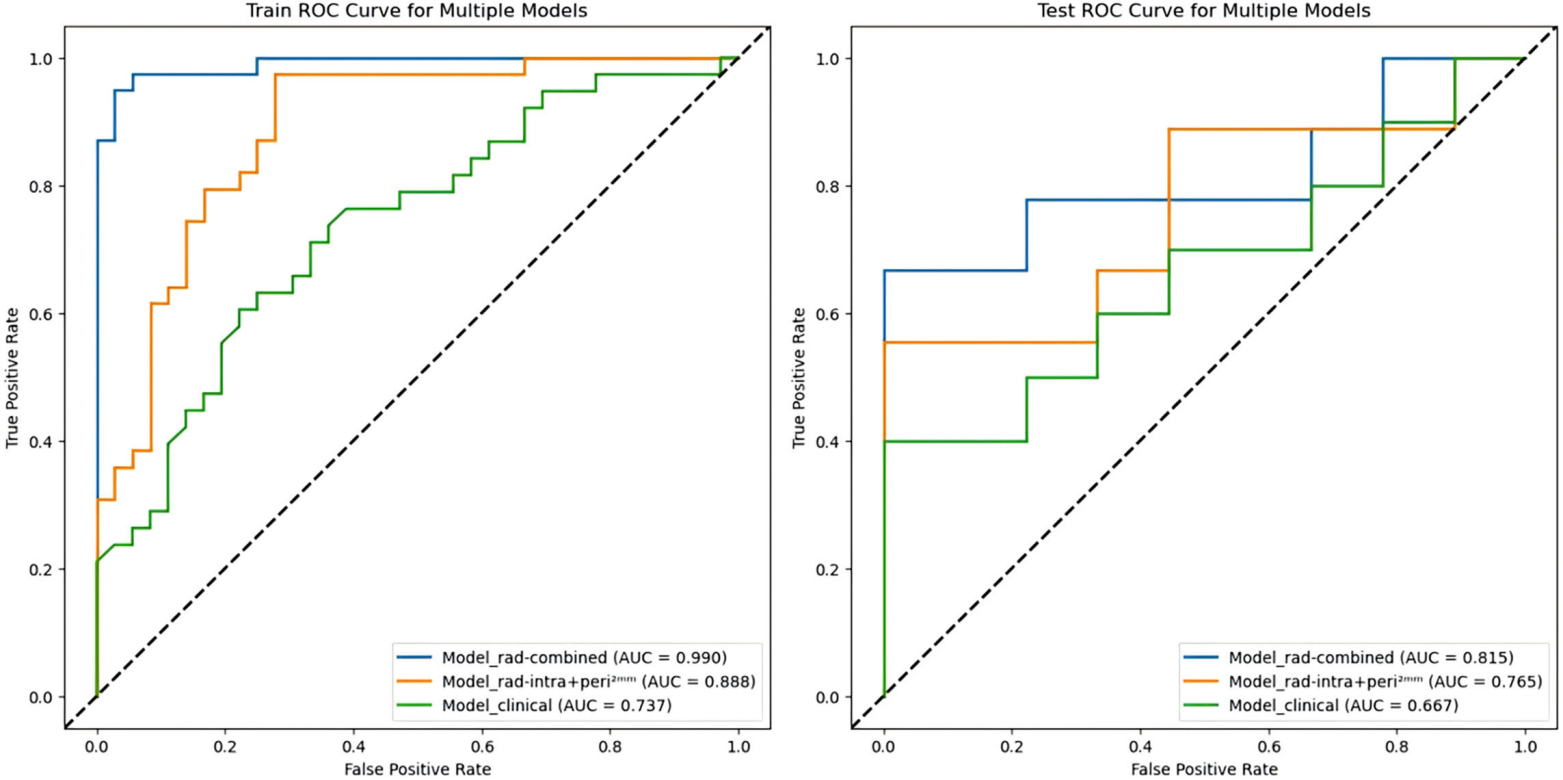
ROC curves of three pGR prediction models in the training and test sets. AUC, area under the curve.

**Figure 5:**
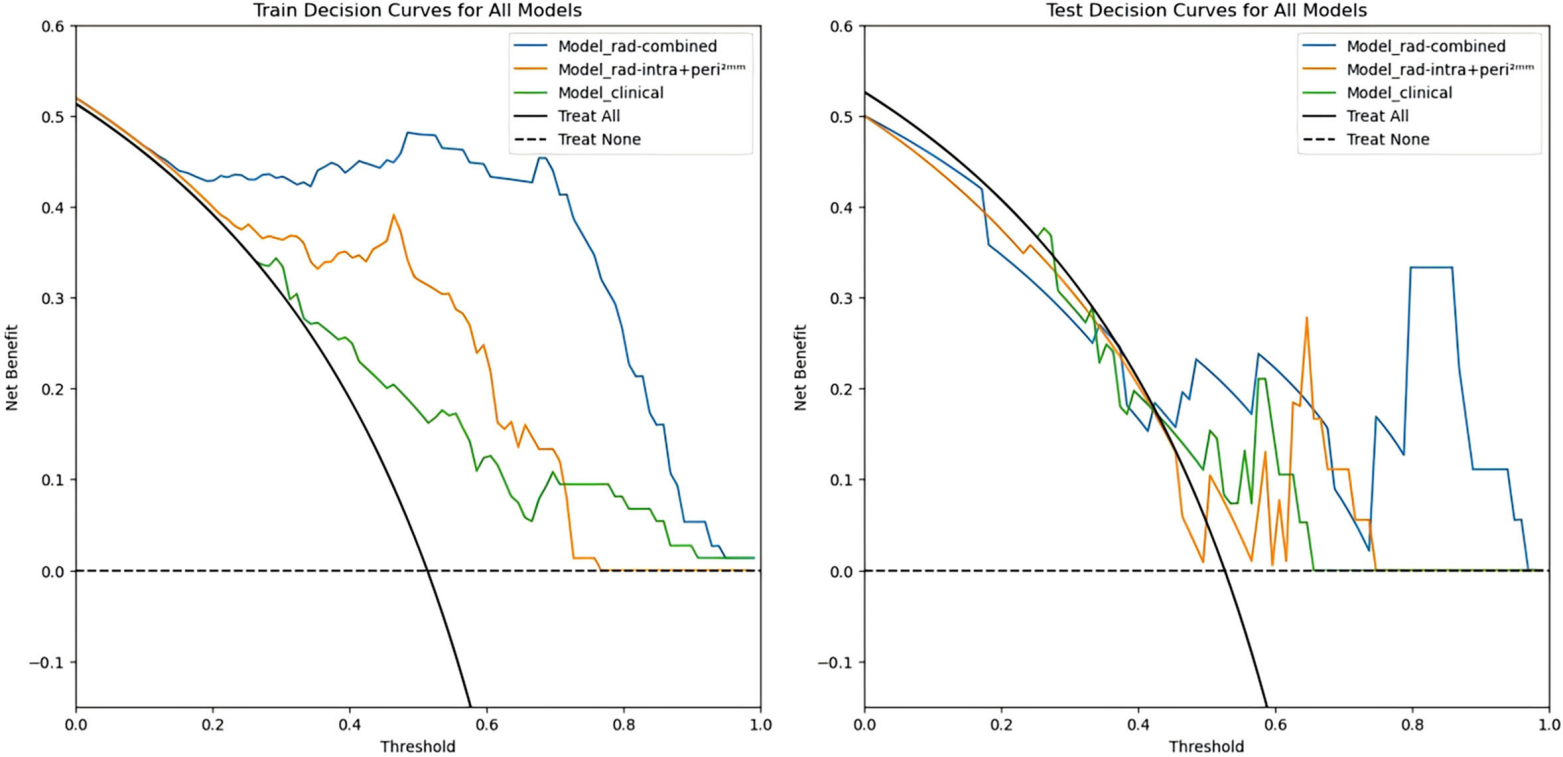
DCA curves of three pGR prediction models in the training set and test set.

**Figure 6:**
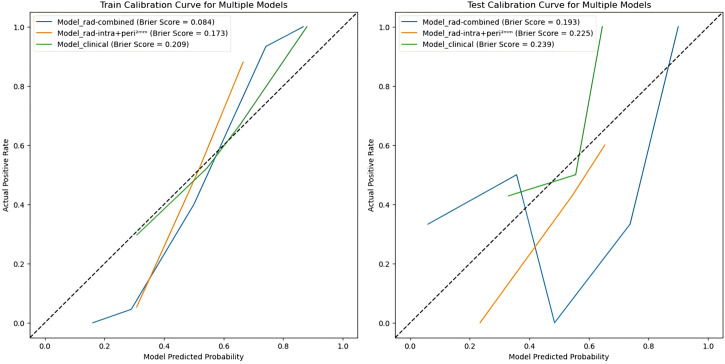
Calibration curves of three pGR prediction models in the training set and test set.

In this study, the radiomics score from the best-performing model, Model_rad-intra + peri^2mm^, was combined with the clinical predictors ALP and PF to construct a nomogram (Model_rad-combined) using multivariate logistic regression analysis (**Figure 3**). This integrated model incorporates both radiomic and clinical features, significantly improving the accuracy and reliability of predicting pGR status. ROC curve analysis (**Figure 4**) showed that the Model_rad-combined model had the best predictive performance, with an AUC of 0.990 (95% CI: 0.975-1.000) in the training set and 0.815 (95% CI: 0.594-1.000) in the test set. The corresponding AUC values for each model are detailed in **Table 5**. DCA curve analysis (**Figure 5**) indicated that Model_rad-combined showed the highest net benefit across a wide range of threshold probabilities. The overall Brier score for the Model_rad-combined was 0.193, indicating a good agreement between the predicted probabilities and the actual outcomes. According to Delong’s tests, in the training set, the AUC of the radiomics nomogram was not significantly different from those of Model rad-combined and Model clinical (*P* = 0.936), Model rad-combined and Model_rad-intra+peri^2mm^ (*P* = 0.695), Model clinical and Model_rad-intra+peri^2mm^ (*P* = 0.645). The calibration curve (**Figure 6**) showed that the prediction curve of Model_rad-combined closely followed the ideal curve, demonstrating optimal calibration performance in each probability interval, with a high degree of alignment between predicted probabilities and actual positive rates, thus confirming the model’s reliability and clinical applicability.

**Table 5:** Diagnostic efficiency of the clinical model, radiomics model and combine model.

Model	AUC (95%CI)	Accuracy	Sensitivity	Specificity	Precision	F1
Training set						
Model_clinical	0.737(0.623-0.850)	0.662	0.763	0.556	0.644	0.699
Model_rad-intra+peri^2mm^	0.888(0.813-0.963)	0.747	0.641	0.861	0.833	0.725
Model_rad-combined	0.990(0.975-1.000)	0.920	0.872	0.972	0.971	0.919
Test set						
Model_clinical	0.667(0.411-0.923)	0.579	0.700	0.444	0.583	0.636
Model_rad-intra+peri^2mm^	0.765(0.528-1.000)	0.667	0.889	0.444	0.615	0.727
Model_rad-combined	0.815(0.594-1.000)	0.778	0.778	0.778	0.778	0.778

### 3.6 Visualization by SHAP

**Figure 7:**
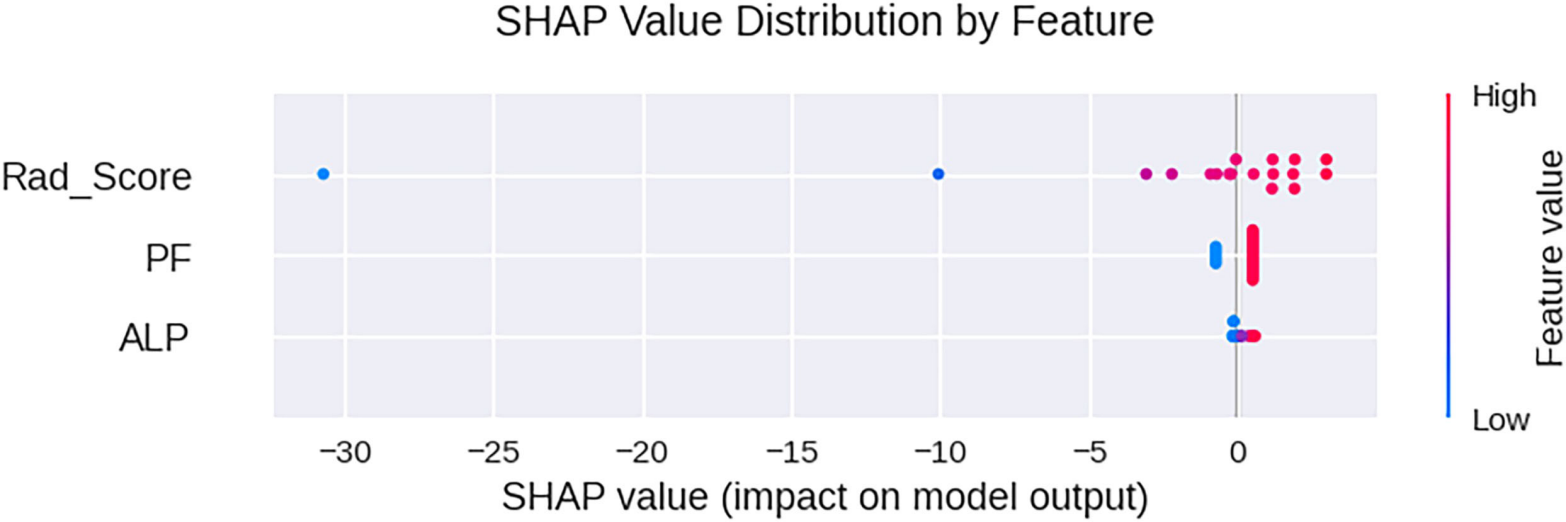
SHAP plot of the nomogram prediction model. The distribution of SHAP values (horizontal axis) for radiomics features (vertical axis), where each point represents a sample. The darker the red color, the larger the feature value; the darker the blue color, the smaller the feature value. Points located to the left of the central vertical line have SHAP values less than zero, which have a negative impact on the prediction; points located to the right of the central vertical line have SHAP values greater than zero, which have a positive impact on the prediction.

The SHAP plot (**Figure 7**) illustrates the relative contributions of the Rad-score, PF, and ALP to the model’s predictions. Rad-score emerged as the most influential feature, exhibiting a broad range of impacts on the output. Higher values of the Rad-score were consistently associated with positive contributions toward predicting pGR. The presence of PF also played a notable role, exerting a positive effect on the model output when it was present. In contrast, ALP showed a relatively limited influence, with SHAP values clustered near zero, indicating a minor overall contribution to the prediction model.

## 4 Discussion

In this study, we developed a combined radiomics-clinical model (Model_rad-combined) to assess pGR in OS patients following NAC, achieving AUCs of 0.990 and 0.815 in the training and test sets, respectively. This model provides a comprehensive evaluation of treatment response and may assist clinicians in identifying patients with poor sensitivity to NAC more effectively. Given that OS is the most common primary malignant bone tumor characterized by high invasiveness and early distant metastasis, with 5-year survival rates dropping below 20% once metastasis occurs (22, 23), early identification of reliable biomarkers for predicting treatment response is crucial for optimizing individualized treatment strategies and improving patient prognosis.

Among the clinical characteristics analyzed, only ALP and PF demonstrated independent predictive value for NAC efficacy. Other variables, including age, sex, primary tumor site, pathological subtype, maximum tumor diameter, presence of distant lung metastasis, and LDH, did not show significant predictive power. These findings are consistent with previous studies employing traditional statistical methods (24, 25). An elevated ALP level may indicate the osteogenic activity of the tumor or the metabolic burden of the liver (26). PF, on the other hand, suggests a strong local invasion and poor bone stability. Both of these factors are associated with a poor response to chemotherapy. The inability of these conventional clinical indicators to demonstrate robust predictive effects may be attributed to several factors: the relatively limited sample size, inherent limitations of single-center data sources, and the confounding influence of regional or population-specific variations. After further integrating the clinical independent predictors (ALP and PF), the combined model (Model_rad-combined) achieved an AUC improvement of 0.815 in the test set, and the decision curve analysis revealed that it had a higher clinical net benefit within a wider threshold range. This improvement indicates that the radiomics features and clinical indicators may have complementary relationships in the prediction mechanism: radiomics captures the heterogeneous imaging phenotypes of tumors and their microenvironments, while ALP and PF reflect the systemic metabolic status and the degree of local bone destruction. The combination of the two can provide a more comprehensive basis for individualized treatment decisions.

In OS, histologic response to NAC is one of the most important prognostic factors, yet it is typically assessed only after surgery, which limits its ability to inform treatment planning before or early during therapy. Radiomics offers a noninvasive strategy to extract high-dimensional quantitative information from routine imaging, enabling a more objective characterization of intratumoral heterogeneity and the tumor microenvironment and potentially improving reproducibility beyond subjective visual interpretation (27). Building on this rationale, we developed a pretreatment T2WI-FS–based radiomics approach for early identification of patients at high risk of chemoresistance. Our best Intratumoral + peritumoral^2mm^ model achieved an AUC of 0.765 in the test set with a sensitivity of 0.889, suggesting utility as a screening tool to minimize false negatives when identifying potentially poor responders. After integrating the radiomics signature with independent clinical predictors (ALP and PF), the final clinical model (Model_rad-combined) improved performance in the test set (AUC = 0.815) with balanced sensitivity and specificity (both 0.778), and demonstrated a higher net benefit on decision-curve analysis, supporting its potential clinical usefulness for pretreatment risk stratification. Importantly, for patients predicted to be at a high risk of chemoresistance, this information can prompt earlier multidisciplinary discussions, shorter intervals for early response reassessment, earlier optimization of local control and surgical planning, and novel therapeutic strategies. These measures aim to reduce the cumulative toxicity and delays associated with ineffective chemotherapy. At the same time, EURAMOS-1 showed that intensifying postoperative chemotherapy for poor histologic responders did not improve outcomes and increased toxicity, indicating that the early identification of resistance should more reasonably support trial allocation and pathway optimization rather than empirical intensification of conventional regimens (28). Conversely, patients predicted to be low risk may proceed with standard therapy while avoiding unnecessary escalation, although these risk-adapted strategies require prospective validation in OS. Notably, radiomics-informed stratification has been explored to guide adaptive treatment decisions in other malignancies, including nasopharyngeal carcinoma and gastric cancer (29, 30). This suggests that it may also be applicable in OS.

Cellular interactions within the tumor microenvironment (TME) play a decisive role in tumor initiation, progression, metastasis, and response to treatment (31, 32). The TME plays a crucial role in facilitating or inhibiting tumor growth and dissemination. The TME as a target for cancer treatment has attracted substantial research and clinical interest. Ding J et al. (33) explored the impact of different widths of the tumor regions around the sentinel lymph nodes in breast cancer on the predictive performance, and found that narrower regions often contained the most relevant biological signals. Given the critical role of the TME in tumor progression and its spatial heterogeneity. This study further compared the predictive efficacy of different peritumoral expansion ranges (2mm, 4mm, 6mm). These widths were selected based on prior surgical evidence establishing 2 mm as a critical margin threshold for local recurrence assessment (34), with wider expansions included to comprehensively characterize peritumoral heterogeneity. Our results demonstrated that Model_rad-intra+peri^2mm^ achieved superior performance in distinguishing pGR status, increasing the test set AUC from 0.728 (intratumoral only) to 0.765. This finding aligns with Duan et al. (35), who similarly reported optimal efficacy with narrower peritumoral ranges. As the tumor expands to 4 mm or 6 mm around the periphery, the tumor-specific signal gradually becomes diluted by normal tissues, and the noise increases. The predictive efficacy then decreases. This suggests that in future studies, when defining the peritumoral area, one should avoid blindly expanding the scope and instead make a refined division based on anatomical and pathological criteria. The superior performance of the 2 mm peritumoral model may reflect that the narrow band immediately adjacent to the tumor boundary represents the most biologically active “critical response zone,” whereas tumor-specific signals decay and become diluted with normal tissue at greater distances, introducing noise that diminishes predictive efficacy. Meanwhile, this result may be related to the spatial specificity of the interaction between the tumor and the microenvironment: the narrow area adjacent to the tumor border often contains pro-inflammatory cells, new blood vessels, and fibroblast interstitial reactions, which may directly affect the permeability of chemotherapy drugs and the local immune status. Previous studies have shown that the histological characteristics within 2mm of the OS margin are significantly associated with the local recurrence risk (34, 36). This study confirmed the key role of this area in predicting treatment responses from the perspective of radiomics. In the future, combined with spatial transcriptomics or microscopic imaging techniques, the corresponding relationship between imaging features and molecular phenotypes within this area can be further revealed.

Through SHAP analysis, it can be seen that the radiomics score (Rad-score) has the greatest contribution to the model output, and its high value tends to predict pGR, which is consistent with the biological nature of the imaging features reflecting the regularity of tumor structure and low heterogeneity. The presence of PF also shows a positive contribution, further confirming its role as an adverse prognostic factor. Although the distribution of ALP’s SHAP value is relatively concentrated, its inclusion still brings a robust improvement in model performance. The nomogram and SHAP visualization provide clinicians with intuitive tools, helping them understand the basis of model decisions, enhancing trust, and promoting its application in multidisciplinary diagnosis and treatment. This model will be applicable in scenarios such as early efficacy prediction, surgical planning assistance, and clinical trial stratification in the future.

Several limitations warrant acknowledgment. First, no *a priori* sample size calculation was performed due to the retrospective design and the rarity of OS, therefore, all consecutive eligible patients were included to minimize selection bias. The modest cohort size may limit generalizability and increase the risk of overfitting, as suggested by the performance gap between the training and test cohorts. We mitigated this risk through feature selection, cross-validation, and evaluation on an independent test set; nevertheless, prospective multicenter studies with larger cohorts are needed for external validation. Second, although all patients received NAC according to standardized National Comprehensive Cancer Network (NCCN) guidelines (adriamycin, methotrexate, cisplatin, ifosfamide), subtle variations in drug administration schedules or individual patient tolerance may exist. We acknowledge that heterogeneity in chemotherapy regimens was not explicitly modeled as a variable, which represents a potential confounding factor that should be addressed in future investigations. Third, this study constructed radiomics models based on a single MRI sequence (T2WI-FS). Existing evidence suggests that multi-sequence MRI analysis can more comprehensively capture tumor heterogeneity, thereby improving model predictive performance. Future studies should incorporate multiple MRI sequences to enhance feature diversity and model accuracy. Finally, although we evaluated peritumoral region models with three different expansion widths (2 mm, 4 mm, and 6 mm), more optimal peritumoral ranges or feature combinations may exist that warrant further exploration. Future research employing finer-scale expansion intervals or machine learning-based optimization techniques for adaptive margin selection could potentially identify superior peritumoral characterization strategies.

## 5 Conclusions

This retrospective cohort study developed a model to predict the efficacy of NAC in OS patients. The model integrates preoperative MR radiomics features with postoperative pathological pGR status and demonstrates strong predictive performance in both the training and test sets. These findings suggest that the model may aid clinicians in early prediction of pGR status in OS patients following NAC, offering valuable guidance for formulating individualized treatment plans.

## Data Availability

The original contributions presented in the study are included in the article/**Supplementary Material**. Further inquiries can be directed to the corresponding authors.
